# *Schistosoma mansoni* and soil-transmitted helminth infections among schoolchildren living along the shore of Lake Hawassa, southern Ethiopia

**DOI:** 10.1186/s13071-024-06578-x

**Published:** 2024-12-03

**Authors:** Belay Beyene, Susana Vaz Nery, Tariku Lambiyo, Techalew Shimelis

**Affiliations:** 1 College of Medicine and Health Sciences, Hawassa University, Hawassa, Ethiopia; 2 Arba Minch College of Health Sciences, Arba Minch, Ethiopia; 3 Kirby Institute, University of New South Wales, Sydney, Australia

**Keywords:** Prevalence, *S. mansoni*, Soil-transmitted helminths, Deworming, Diagnostic methods

## Abstract

**Background:**

*Schistosoma mansoni* and soil-transmitted helminth (STH) infections are major public health problems in areas with poor sanitation and limited access to water. In Ethiopia, there is limited data available for monitoring the efficacy of interventions aimed at reducing helminth infections. Therefore, we assessed the prevalence of *S. mansoni* and STH infections, as well as factors associated with this prevalence, among schoolchildren and compared the findings with those of earlier studies. We also evaluated the diagnostic agreement between two parasitological methods.

**Methods:**

A cross-sectional study involving 363 schoolchildren from three rural primary schools located along the shore of Lake Hawassa, Sidama Regional State, southern Ethiopia, was conducted in October and November 2023. The schoolchildren were selected using a systematic random sampling technique. Socio-demographic data were collected using pre-structured questionnaires. A single stool sample was collected from each study participant and processed using direct wet mount (DM) microscopy and the formol-ether concentration technique (FECT) to detect helminth ova.

**Results:**

The overall prevalence of helminths was 59.8%, with 36.6% of participating children having a single infection and 23.1% having multiple infections. *Schistosoma mansoni* and STHs were present in 33.9% and 38.8% of children, respectively. The STHs included *Ascaris lumbricoides* (28.9% of children), *Trichuris trichiura* (10.7%), hookworms (5.2%) and *Strongyloides stercoralis* (2.8%). Diagnostic agreement between the DM microscopy method and FECT was substantial [kappa (*κ*) = 0.710] for the detection of *Hymenolepis nana* and almost perfect (κ = 0.827) for the dection of *A. lumbricoides*, but only fair for the detection of other detected helminths. Children at Finchawa primary school had a lower prevalence of *S. mansoni* infection [adjusted odds ratio (AOR) 0.31; 95% confidence interval (CI) 0.13–0.76] than those at St Paul’s Tullo Catholic primary school. STH infections were more common among children who sometimes (vs. always) washed their hands before meals (AOR 1.89; 95% CI 1.01–3.54) and those who regularly played with soil (AOR 2.56; 95% CI 1.47–4.46).

**Conclusions:**

This study showed a reduction in STH infections from a high prevalence in 2015 to a moderate prevalence at the present time, despite a similar moderate prevalence of *S. mansoni* infection. Thus, it is crucial to intensify deworming interventions to reduce the burden of helminths in the study area. Additionally, there is a need to enhance the capacity of clinical laboratories to perform FECT in Ethiopian clinical settings where DM is often employed to diagnose helminths.

**Graphical Abstract:**

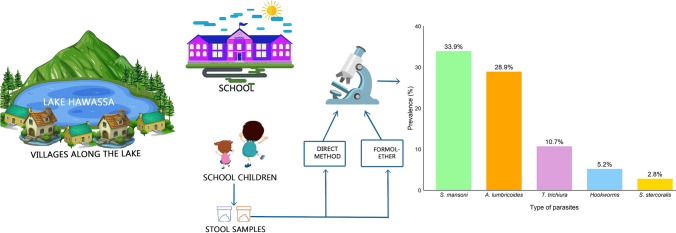

**Supplementary Information:**

The online version contains supplementary material available at 10.1186/s13071-024-06578-x.

## Background

Soil-transmitted helminths (STHs) remain a significant global health issue, particularly in locations where residents have limited access to clean water, poor sanitation and inadequate hygiene practices. An estimated 1.5 billion people worldwide are infected with STHs, including 654 million school-aged children living in areas where these parasites are prevalent [1]. *Ascaris lumbricoides*, *Trichuris trichiura*, and hookworms are the most common STHs in tropical and subtropical areas, with the highest health burden in sub-Saharan Africa [1]. Infection with *Schistosoma mansoni* also poses a major public health concern, with over 250 million people infected worldwide [2].

Infections with *A. lumbricoides* and *T. trichiura* typically occur through the ingestion of food, water or soil contaminated with infective eggs. Hookworm and *S. mansoni* infections are contracted through skin penetration by infectious larvae found in contaminated soil and water bodies, respectively [1, 2]. Children are particularly at higher risk for helminth infections due to poor hygiene practices and behaviors such as walking barefoot, playing in soil and swimming in contaminated water bodies [3]. The morbidity associated with STH and *S. mansoni* infections in children includes anemia, malnutrition, stunted growth, cognitive impairments, decreased school attendance and poor academic performance [4].

In Ethiopia, an estimated 79 million people and 37.3 million people live in areas endemic for STHs and *S. mansoni*, respectively, with the majority of these being school-aged children, totaling 25.3 million and 12.3 million children, respectively [5]. As part of the Enhanced Outreach Strategy, the Federal Ministry of Ethiopia has been providing deworming interventions for STHs targeting preschool-aged children since 2004, alongside implementing water, sanitation and hygiene (WASH) programs [6, 7]. Deworming efforts for school-aged children that addressed both STHs and *S. mansoni* began in 2007 [7]. The national mapping surveys conducted between 2013 and 2015 throughout Ethiopia's regional states showed a prevalence of 21.7% for STHs and 3.5% for *S. mansoni* infections among school-aged children, with significant regional variation [8]. In 2015, Ethiopia launched a national control program aimed at eliminating *S. mansoni* and STHs as significant public health problems by 2020 and breaking transmission by 2025 [7]. The program’s goals include treating at least 75% of school-aged children, extending treatment to adolescents and adults, integrating neglected tropical disease programs and enhancing collaboration between mass drug administration and WASH initiatives [5].

Results from systematic reviews have demonstrated that these helminth control interventions have significantly reduced the prevalence of STH and *S. mansoni* infections over decades in Ethiopia [7, 9]. Similarly, a survey of schoolchildren in our study area showed a marked decline in the prevalence of STHs (52.4%) and *S. mansoni* (31%) in 2015 [10] as compared to STHs (67.3%) and *S. mansoni* (76.5%) in 2007 [11]. However, the current status of these infections in the study area following the scale-up of the helminth control interventions is unknown.

Direct wet-mount (DM) microscopy is widely used in Ethiopian clinical settings to diagnose helminth infections due to its ease of use and rapid results [12]. However, DM microscopy is less sensitive than other parasitological methods, such as the Kato-Katz (KK) and formol-ether concentration (FECT) techniques. For example, a study conducted in a similar helminth prevalence setting in Ethiopia reported that the DM method had sensitivities of 22.1% for *S. mansoni*, 52.0% for *A. lumbricoides*, 12.5% for *T. trichiura* and 37.9% for hookworms; in comparison, the FECT showed sensitivities of 58.4%, 81.4%, 57.8% and 72.4%, respectively, while the KK method had sensitivities of 96.1%, 93.1%, 90.6% and 69.0% for the same parasites, respectively [13]. A similar sensitivity between FECT and KK has also been reported by others based on a single stool sample for detecting helminths [14, 15]. The lower sensitivity of DM microscopy often results in misdiagnosis, particularly for helminths present in lower concentrations, leading to inappropriate clinical interventions [13].

This study aimed to determine the prevalence of *S. mansoni* and STH infections, as well as the factors associated with prevalence, among schoolchildren living along the shore of Lake Hawassa, in order to evaluate the progress of ongoing control interventions. We also investigated the diagnostic agreement between DM microscopy and the FECT.

## Methods

### Study design and setting

**Fig. 1:**
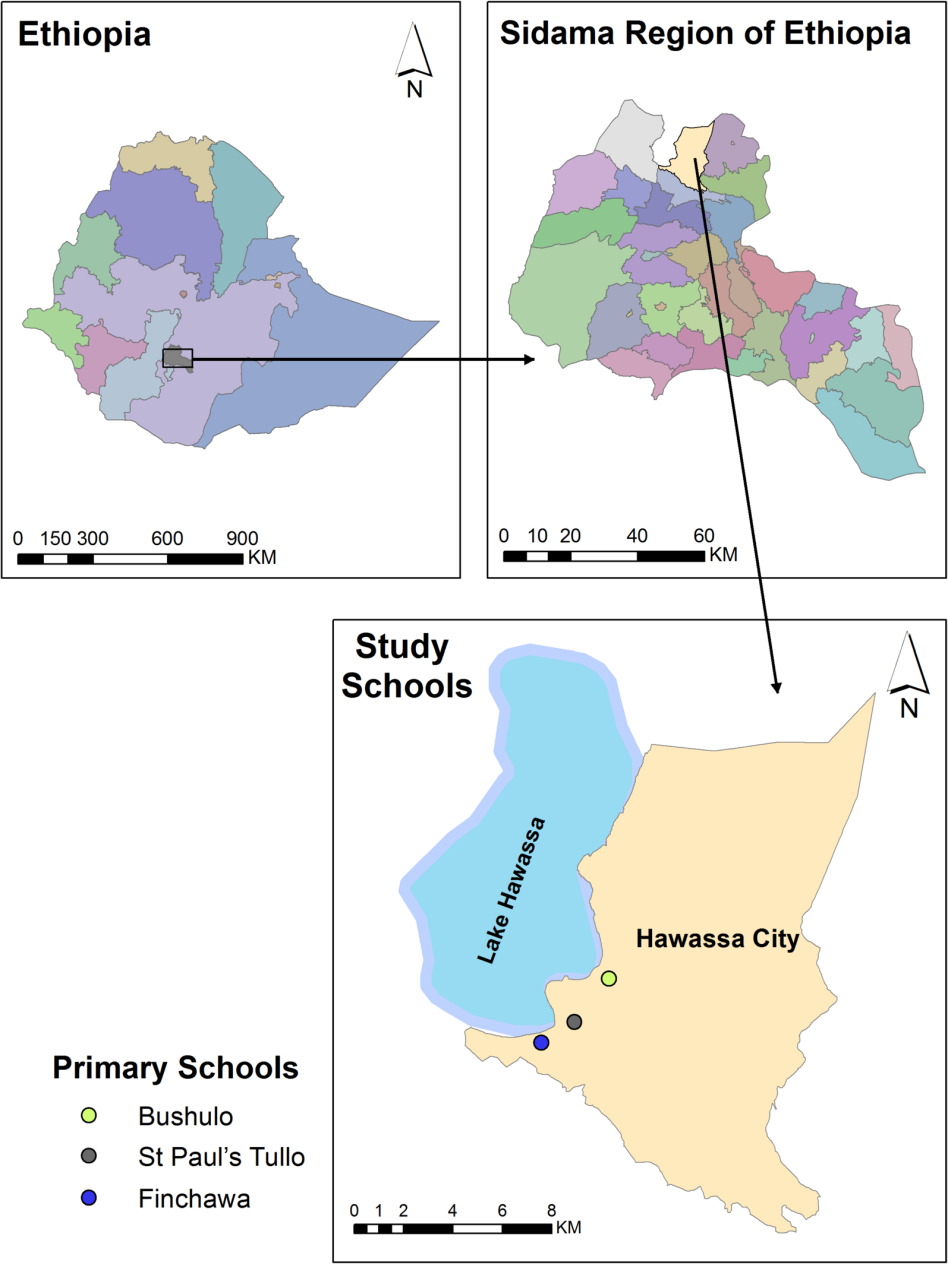
Map of the study sites, Sidama Region, southern Ethiopia, 2023. Prepared using ArcGIS 10.8 (ESRI, Redlands, CA USA) after obtaining country shape files from the humdata.org website (https://data.humdata.org/)

A cross-sectional study was conducted in October and November 2023 among schoolchildren attending three primary schools: Finchawa, St Paul’s Tullo Catholic (PTC) and Bushulo. These schools are located in Finchawa and Tullo (PTC and Bushulo schools, respectively) *kebeles* (the smallest administrative units in Ethiopia) in the Hawella-Tula sub-city of Hawassa, the capital of Sidama Regional State. PTC school is operated by a non-governmental organization, and Finchawa and Bushulo are public schools. The Hawella-Tula sub-city has 11 rural and 1 urban *kebeles,* whereas the other seven sub-cities of Hawassa City have only urban *kebeles*. Finchewa and Tullo are two of the 11 rural kebeles bordering Lake Hawassa, with a total population of 145,831 (74,795 males and 71,036 females). The lake is primarily used by the inhabitants for household needs, recreation, fishing and laundry. The three schools were selected specifically due to their proximity to the lake (Fig. 1), where *S. mansoni* infection is prevalent, and due to the availability of baseline data from similar surveys conducted in 2015 and 2007 [10, 11].

Hawassa (7°3′43.38″N, 38°28′34.86″E) is located 270 km south of Addis Ababa, at an altitude of 1680 m a.s.l. The mean annual temperature is 20.9 °C, and mean annual rainfall is 997.6 mm. Hawella-Tula sub-city has 27 health facilities and 69 schools, of which 15 offer grades 1–8 [16]. According to information gathered orally from the sub-city health office on existing interventions as of 2015, the STH deworming program for school-aged children has been consistently conducted biannually. However, preventive chemotherapy for *S. mansoni* infection was only administered during the first 2 years (2015 and 2016) of the program due to a scarcity of praziquantel, an anthelmintic medication used to treat *S. mansoni* infections.

### Population

All schoolchildren aged 6 to 16 years attending the selected schools during the study period were enrolled in the study. Students who volunteered to participate and confirmed that they had not taken anti-helminthic drugs within 1 month prior to the time of data collection were included.

### Sample size and sampling technique

The sample size was computed using a single population proportion formula, assuming a prevalence of *S. mansoni* infection of 31% [10], a 95% level of confidence and a 5% margin of error. After accounting for a 10% non-respondent rate, the sample size was determined to be 363 children.

The number of children from each of the three schools included in the study (sample size) was based on each school's proportion of the total number of students in the three schools (Finchewa, *n* = 523 children; PTC, *n* = 547; Bushulo, *n* = 1895). Similarly, the number of students enrolled from each grade was determined proportionally. The participants from each grade were selected using a systematic random sampling technique, with class rosters serving as the sampling frame. The *k*th interval was calculated by dividing the total number of students (*N* = 2965) in the three schools by the sample size (*n* = 363), resulting in a *k*th value of 8 (2965/363). Thus, at every eighth interval, 64 students from Finchawa, 67 students from PTC and 232 students from Bushulo were chosen to give stool samples. The starting point in the sampling frame was chosen by a lottery method.

### Data collection methods

#### Interview

Data on socio-demographic, behavioral and environmental factors were collected by medical laboratory technologists with Bachelor’s degrees in medical laboratory technology. Data collectors visited each student’s home, interviewed parents/guardians and collected information using paper-based questionnaires.

#### Laboratory investigation

About 5 g of a stool sample was collected from each participant by medical laboratory technologists using clean stool collection cups. Samples were labeled using unique identification numbers, placed in a triple packaging system and transported to the laboratory at the College of Medicine and Health Sciences, Hawassa University for analyses within 1 h of collection. The samples were processed using DM microscopy and FECT [17] and examined for ova/larva of parasites by the medical laboratory technologists. One laboratory technologist performed DM microscopy and another performed FECT, with each examining two slides from each stool sample. The readers were blinded to each other’s results. A composite reference standard was used, where the detection of a parasite by either DM microscopy or FECT was interpreted as a positive result and considered to assess the prevalence.

### Data analysis

The collected data were double-entered into EpiData software version 4.6 and analyzed using SPSS version 25 (SPSS IBM Corp. Armonk, NY, USA). The results of analyses on qualitative variables (e.g., sex, family occupation and distribution of helminths) were summarized using frequencies and percentages, while the results of analyses on continuous variables (e.g., child’s age) were summarized using means [± standard deviation (SD)] and ranges. The crude odds ratios (COR) from the binary logistic regression analysis were used to evaluate the association between independent variables (socio-demographic, behavioral and environmental factors) and *S. mansoni* and STH infections. The adjusted odds ratios (AOR) were calculated using multivariable logistic regression analysis, including all variables with a *p*-value < 0.25 in the bivariate analysis. A *p*-value < 0.05 was considered to be statistically significant. Diagnostic agreements between DM microscopy and FECT were assessed using Cohen’s kappa (*κ*) value at a 95% confidence interval (CI). The *κ* results were interpreted as follows: values ≤ 0 as no agreement; 0.01–0.20 as slight agreement; 0.21–0.40 as fair agreement; 0.41–0.60 as moderate agreement; 0.61–0.80 as substantial agreement; and 0.81–1.00 as almost perfect agreement [18].

## Results

### Socio-demographic characteristics of the study participants

A total of 363 schoolchildren participated in the study, with 63.9% of the participating schoolchildren from Bushulo school, 17.6% from Finchawa school and 18.5% from PTC school. The mean (± SD) age of the study participants was 11.3 ± 3.1 (range, 6–16) years. The proportions of participants in the age group 13–16 years accounted for 41.9% of participating children, followed by 32.5% in the age group 6–9 years. The male-to-female ratio was 0.78:1. Among the participants, 48.5% had fathers who were farmers, and 66.4% had mothers who were housewives.

### Prevalence of *S. mansoni* and STH infections

Of the 363 study participants, 59.8% (95% CI 54.5–64.9) were found to harbor at least one helminth parasite. *Schistosoma mansoni* infection was found in 33.9% (95% CI 29.0–39.0) of the children. The prevalence of infections with STHs was 38.8% (95% CI 33.8–44.1), with *A. lumbricoides*, *T. trichiura*, hookworm and *Strongyloides stercoralis* infections found in 28.9% (95% CI 24.3–33.9), 10.7% (95% CI 7.8–14.4), 5.2% (95% CI 3.2–8.1) and 2.8% (95% CI 4.7–10.3) of the children, respectively. Other parasites detected were *Hymenolepis nana* (4.4%; 95% CI 2.5–7.1), *Enterobius vermicularis* (2.2%; 95% CI 1.0–4.3) and *Taenia* species (1.1%; 95% CI 0.3–2.8).

In this study, 36.6% (95% CI 31.7–41.8) of study participants had single infections, and 23.1% (95% CI 18.9–27.8) had co-infections (> 1 type of parasite). The prevalence of double, triple and quadruple parasitic infections among schoolchildren was 17.6%, 4.7% and 0.8%, respectively. Dual infections with *A. lumbricoides* and *S. mansoni* (7.4%) were the most frequently observed double infections (Table 1).

**Table 1:** Distribution of single and co-infections among schoolchildren attending the three primary schools in Hawassa, southern Ethiopia, 2023

Parasite	Frequency (*n* schoolchildren)	Percentage
Single infection	133	36.6
*Schistosoma mansoni* only	64	17.6
*Ascaris lumbricoides* only	45	12.4
*Trichuris trichiura* only	9	2.5
*Strongyloides stercoralis* only	4	1.1
Hookworm only	4	1.1
*Enterobius vermicularis* only	3	0.8
*Hymenolepis nana* only	3	0.8
*Taenia* species only	1	0.3
Double infections	64	17.6
*A. lumbricoides* and *S. mansoni*	27	7.4
*S. mansoni* and *T. trichiura*	10	2.8
*A. lumbricoides* and *T. trichiura*	8	2.2
*A. lumbricoides* and *S. stercoralis*	3	0.8
*S. mansoni* and *H. nana*	3	0.8
*S. mansoni* and hookworm	3	0.8
*A. lumbricoides* and hookworm	2	0.6
*T. trichiura* and hookworm	1	0.3
*A. lumbricoides* and *H. nana*	1	0.3
*A. lumbricoides* and *Taenia* species	1	0.3
*A. lumbricoides* and *E. vermicularis*	1	0.3
*S. mansoni* and *E. vermicularis*	1	0.3
*S. stercoralis* and *T. trichiura*	1	0.3
*T. trichiura* and *H. nana*	1	0.3
*H. nana* and *E. vermicularis*	1	0.3
Triple infections	17	4.7
Hookworm, *A. lumbricoides* and *S. mansoni*	4	1.1
*A. lumbricoides, S. mansoni* and *H. nana*	3	0.8
*A. lumbricoides*, *T. trichiura* and *S. mansoni*	2	0.6
Hookworm, *A. lumbricoides* and *T. trichiura*	2	0.6
*S. mansoni*, *A. lumbricoides* and *S. stercoralis*	1	0.3
Hookworm, *A. lumbricoides* and *H. nana*	1	0.3
*T. trichiura, E. vermicularis* and *H. nana*	1	0.3
*S. stercoralis,* Taenia species and *H. nana*	1	0.3
*A. lumbricoides, S. mansoni* and Taenia species	1	0.3
Quadruple infection	3	0.8
*A. lumbricoides, S. mansoni*, hookworm and *T. trichiura*	2	0.6
*A. lumbricoides, T. trichiura, E. vermicularis* and *S. mansoni*	1	0.3
No helminthic infection	146	40.2

### Distribution of helminth infections by socio-demographic and other factors

Detailed findings on the distribution of helminth infections by socio-demographic and other factors are presented in Tables 2 and 3. The multivariable logistic regression analysis showed that children attending Finchawa school had significantly lower odds of *S. mansoni* infection (AOR 0.31; 95% CI 0.13–0.76) than children attending PTC school. Additionally, children who sometimes washed their hands after latrine use were more likely to have *S. mansoni* infection (AOR 2.45; 95% CI 1.19–5.04) than those who always washed their hands (Table 2). The odds of having STH infections were significantly higher in children who sometimes washed their hands before meals (AOR 1.89; 95% CI 1.01–3.54) compared to those who always did so. Similarly, children with a habit of playing with soil had higher odds of STH infections (AOR 2.56; 95% CI 1.47–4.46) than those who did not commonly play with soil (Table 3).

**Table 2:** Distribution of *Schistosoma mansoni* infection by socio-demographic and other factors associated with infection among schoolchildren in Hawassa, southern Ethiopia, 2023

Variables	*Schistosoma mansoni* infection			COR (95% CI)	*p*-value	AOR (95% CI)	*p*-value
	Total (*N* = 363), *n* (%)	Positive, *n* (%)	Negative, *n* (%)				
*Age category (year)*							
6–9	118 (32.5)	36 (30.5)	82 (69.5)	1		1	
10–12	93 (25.6)	38 (40.9)	55 (59.1)	1.57 (0.89–2.78)	0.119	1.87 (1.00–3.51)	0.051
13–16	152 (41.9)	49 (32.2)	103 (67.8)	1.08 (0.65–1.82)	0.762	1.44 (0.79–2.56)	0.223
*Sex*							
Male	159 (43.8)	57 (35.8)	102 (64.2)	1		-	
Female	204 (56.2)	66 (32.4)	138 (67.6)	0.86 (0.55–1.33)	0.485		
*Child's school*							
Bushulo	232 (63.9)	83 (35.8)	149 (64.2)	0.83 (0.48–1.44)	0.499	1.09 (0.56–2.12)	0.800
Finchawa	64 (17.6)	13 (20.3)	51 (79.7)	0.38 (0.17–0.82)	0.014	0.31 (0.13–0.76)	0.011*
PTC	67 (18.5)	27 (40.3)	40 (59.7)	1		1	
*Family size*							
2–4	70 (19.3)	22 (31.4)	48 (68.6)	1		–	
5–9	293 (80.7)	101 (34.5)	192 (65.5)	1.15 (0.66–1.01)	0.629		
*Father’s occupation*							
Merchant	85 (23.4)	27 (31.8)	58 (68.2)	1		1	
Farmer	176 (48.5)	60 (34.1)	116 (65.9)	1.11 (0.64–1.93)	0.709	0.85 (0.45–1.61)	0.627
Government employee	58 (16.0)	19 (32.8)	39 (67.2)	1.05 (0.51–2.14)	0.901	1.43 (0.65–3.14)	0.370
Private employee	31 (8.5)	15 (48.4)	16 (51.6)	2.01 (0.87–4.66)	0.102	2.18 (0.84–5.62)	0.108
Others	13 (3.6)	2 (15.4)	11 (84.6)	0.39 (0.08–1.89)	0.242	0.41 (0.08–2.12)	0.295
*Mother’s occupation*							
Merchant	57 (15.7)	15 (26.3)	42 (73.7)	1		1	
Farmer	31 (8.5)	14 (45.2)	17 (54.8)	2.31 (0.92–5.79)	0.075	2.07 (0.70–6.13)	0.189
Government employee	16 (4.4)	2 (12.5)	14 (87.5)	0.40 (0.08–1.97)	0.260	0.52 (0.09–2.88)	0.454
Private employee	14 (3.9)	6 (42.9)	8 (57.1)	2.10 (0.63–7.05)	0.230	2.11 (0.58–7.73)	0.258
Housewife	241 (66.4)	85 (35.3)	156 (64.7)	1.53 (0.80–2.91)	0.200	1.96 (0.96–3.99)	0.066
Other	4 (1.1)	1 (25.0)	3 (75.0)	0.93 (0.09–9.68)	0.954	1.43 (0.12–17.4)	0.777
*Latrine availability at home*							
Yes	324 (89.3)	107 (33.0)	217 (67.0)	1		–	
No	39 (10.7)	16 (41.0)	23 (59.0)	1.41 (0.72–2.78)	0.320		
*Defecation site*							
Latrine	322 (88.7)	109 (33.9)	213 (66.1)	1		–	
Open field	41 (11.3)	14 (34.1)	27 (65.9)	0.99 (0.50–1.96)	0.970		
*Hand-washing before meals*							
Always	146 (40.2)	47 (32.2)	99 (67.8)	1		–	
Sometimes	217 (59.8)	76 (35.0)	141 (65.0)	1.14 (0.73–1.77)	0.576		
*Hand-washing after defecation*							
Always	110 (30.3)	28 (25.5)	82 (74.5)	1		1	
Sometimes	253 (69.7)	95 (37.5)	158 (62.5)	1.76 (1.07–2.90)	0.026	2.45 (1.19–5.04)	0.015*
*Proper latrine use by child*							
Always	194 (53.4)	66 (34.0)	128 (66.0)	1		–	
Sometimes	169 (46.6)	57 (33.7)	112 (66.3)	0.99 (0.64–1.53)	0.953		
*Proper latrine use by family members*							
Always	210 (57.9)	72 (34.3)	138 (65.7)	1		–	
Sometimes	153 (42.1)	51 (33.3)	102 (66.7)	0.96 (0.62–1.49)	0.850		
C*hild was involved in agriculture*							
Yes	191 (52.6)	72 (37.7)	119 (62.3)	1.44 (0.93–2.23)	0.107	1.29 (0.75–2.24)	0.362
No	172 (47.4)	51 (29.7)	121 (70.3)	1		1	
*Child played regularly with soil*							
Yes	81 (22.3)	33 (40.7)	48 (59.3)	1.47 (0.88–2.44)	0.140	1.29 (0.71–2.35)	0.398
No	282 (77.7)	90 (31.9)	192 (68.1)	1		1	
*Main water source*							
Pipe	240 (66.1)	75 (31.2)	165 (68.8)	1		1	
Well	21 (5.8)	8 (38.1)	13 (61.9)	1.35 (0.54–3.40)	0.520	0.84 (0.29–2.42)	0.739
Lake	102 (28.1)	40 (39.2)	62 (60.8)	1.42 (0.87–3.00)	0.155	1.34 (0.74–2.43)	0.342
*Child wearing shoes during data collection*							
Yes	354 (97.5)	118 (33.3)	236 (66.7)	1		1	
No	9 (2.5)	5 (55.6)	4 (44.4)	2.50 (0.66–9.48)	0.178	2.02 (0.48–8.51)	0.340
*Frequency of shoe-wearing habit*							
Always	97 (26.7)	28 (28.9)	69 (71.1)	1		1	
Sometimes	266 (73.3)	95 (35.7)	171 (64.3)	1.37 (0.83–2.27)	0.223	1.05 (0.53–2.11)	0.883
*Type of shoes*							
Open	343 (94.5)	119 (34.7)	224 (65.3)	2.13 (0.69–6.50)	0.019	1.87 (0.56–6.28)	0.308
Closed	20 (5.5)	4 (20.0)	16 (80.0)	1		1	

*AOR* Adjusted odds ratio,* CI* confidence interval, *COR* crude odds ratio, *PTC* St Paul’s Tullo Catholic school

*Significant difference at *p*-value < 0.05

**Table 3:** The distribution of soil-transmitted helminth infections by socio-demographic and other factors associated with infection among schoolchildren in Hawassa, southern Ethiopia, 2023

Variables	Soil-transmitted helminth infections			COR (95% CI)	*p-*value	AOR (95% CI)	*p-*value
	Total (*N* = 363), *n* (%)	Positive, *n* (%)	Negative, *n* (%)				
*Age (year)*							
6–9	118 (32.5)	54 (45.8)	64 (54.2)	1.94 (1.18–3.21)	0.009	1.38 (0.79–2.41)	0.256
10–12	93 (25.6)	41 (44.1)	52 (55.9)	1.82 (1.06–3.11)	0.029	1.67 (0.94–2.95)	0.078
13–16	152 (41.9)	46 (30.3)	106 (69.7)	1		1	
*Sex*							
Male	159 (43.8)	64 (40.3)	95 (59.7)	1		–	
Female	204 (56.2)	77 (37.7)	127 (62.3)	0.90 (0.59–1.38)	0.630		
*Child's school*							
Bushulo	232 (63.9)	93 (40.1)	139 (59.9)	1.20 (0.68–2.11)	0.529	–	
Finchawa	64 (17.6)	24 (37.5)	40 (62.5)	1.08 (0.53–2.19)	0.842		
PTC	67 (18.5)	24 (35.8)	43 (64.2)	1			
*Family size*							
2–4	70 (19.3)	30 (42.9)	40 (57.1)	1		–	
5–9	293 (80.7)	111 (37.9)	182 (62.1)	0.81 (0.48–1.38)	0.440		
*Father’s occupation*							
Merchant	85 (23.4)	28 (32.9)	57 (67.1)	1		1	
Farmer	176 (48.5)	73 (41.5)	103 (58.5)	1.44 (0.84–2.48)	0.186	1.28 (0.72–2.27)	0.409
Government employee	58 (16.0)	25 (43.1)	33 (56.9)	1.54 (0.77–3.07)	0.218	2.01 (0.97–4.17)	0.061
Private employee	31 (8.5)	11 (35.5)	20 (64.5)	1.12 (0.47–2.66)	0.798	1.17 (0.46–2.98)	0.746
Other	13 (3.6)	4 (30.8)	9 (69.2)	0.91 (0.26–3.20)	0.876	0.76 (0.19–2.93)	0.685
*Mother’s occupation*							
Merchant	57 (15.7)	21 (36.8)	36 (63.2)	1		–	
Farmer	31 (8.5)	15 (48.4)	16 (51.6)	1.61 (0.66–3.90)	0.294		
Government employee	16 (4.4)	6 (37.5)	10 (62.5)	1.03 (0.33–3.24)	0.962		
Private employee	14 (3.9)	5 (35.7)	9 (64.3)	0.95 (0.28–3.22)	0.937		
Housewife	241 (66.4)	93 (38.6)	148 (61.4)	1.08 (0.59–1.96)	0.807		
Other	4 (1.1)	1 (25.0)	3 (75.0)	0.57 (0.06–5.58)	0.637		
*Latrine availability at home*							
Yes	324 (89.3)	120 (37.0)	204 (63.0)	1		1	
No	39 (10.7)	21 (53.8)	18 (46.2)	1.98 (1.02–3.87)	0.045	1.52 (0.32–7.22)	0.599
*Defecation site*							
Latrine	322 (88.7)	120 (37.3)	202 (62.7)	1		1	
Open field	41 (11.3)	21 (51.2)	20 (48.8)	0.57 (0.29–1.09)	0.087	1.02 (0.23–4.60)	0.980
Hand-washing before meals							
Always	146 (40.2)	44 (30.1)	102 (69.9)	1		1	
Sometimes	217 (59.8)	97 (44.7)	120 (55.3)	1.87 (1.20–2.92)	0.006	1.89 (1.01–3.54)	0.047*
*Hand-washing after defecation*							
Always	110 (30.3)	34 (30.9)	76 (69.1)	1		1	
Sometimes	253 (69.7)	107 (42.3)	146 (57.7)	1.64 (1.02–2.64)	0.042	0.91 (0.45–1.84)	0.802
*Proper latrine use by child*							
Always	194 (53.4)	64 (33.0)	130 (67.0)	1		1	
Sometimes	169 (46.6)	77 (45.7)	92 (54.4)	1.70 (1.11–2.60)	0.015	1.78 (0.86–3.68)	0.120
*Proper latrine use by family members*							
Always	210 (57.9)	76 (36.2)	134 (63.8)	1		1	
Sometimes	153 (42.1)	65 (42.5)	88 (57.5)	1.30 (0.85–2.00)	0.225	0.68 (0.34–1.37)	0.276
*Child was involved in agriculture*							
Yes	191 (52.6)	78 (40.8)	113 (59.2)	0.84 (0.55–1.28)	0.411	–	
No	172 (47.4)	63 (36.6)	109 (63.4)	1			
*Child played regularly with soil*							
Yes	81 (22.3)	44 (54.3)	37 (45.7)	2.27 (1.37–3.75)	0.001	2.56 (1.47–4.46)	0.001*
No	282 (77.7)	97 (34.4)	185 (65.6)	1		1	
*Main water source*							
Pipe	240 (66.1)	95 (39.6)	145 (60.4)	1		–	
Well	21 (5.8)	8 (38.1)	13 (61.9)	0.94 (0.38–2.35)	0.890		
Lake	102 (28.1)	38 (37.3)	64 (62.7)	0.91 (0.56–1.46)	0.686		
*Child wearing shoes during data collection*							
Yes	354 (97.5)	137 (38.7)	217 (61.3)	1		–	
No	9 (2.5)	4 (44.4)	5 (55.6)	1.27 (0.33–4.80)	0.728		
*Frequency of shoe-wearing habit*							
Always	97 (26.7)	33 (34.0)	64 (66.0)	1		–	
Sometimes	266 (73.3)	108 (40.6)	158 (59.4)	1.33 (0.82–2.16)	0.256		
*Type of shoes*							
Open	343 (94.5)	135 (39.4)	208 (60.6)	1.51 (0.57–4.04)	0.407	–	
Closed	20 (5.5)	6 (30.0)	14 (70.0)	1			

*AOR* Adjusted odds ratio,* CI* confidence interval, *COR* crude odds ratio, *PTC* St Paul’s Tullo Catholic school

*Significant difference at *p*-value < 0.05

The species-wise distribution of STH infections by demographic characteristics is presented in Table 4. The prevalence of *A. lumbricoides, T. trichiura,* hookworm and *S. stercoralis* infections was similar across different age groups, genders and schools.

**Table 4:** Prevalence of *Schistosoma mansoni* and soil-transmitted helminth infections by age, gender and study site at the three rural primary schools in Hawassa, southern Ethiopia, 2023

Characteristics	Total tested, *n* (%)	*Ascaris lumbricoides,* *n* [% (95% CI)]	*Trichuris trichiura*, *n* [% (95% CI)]	Hookworms, *n* [% (95% CI)]	*Strongyloides stercoralis*, *n* [% (95% CI)]
*Age (years)*					
6–9	118 (32.5)	41 [34.7 (26.2–44.1)]	15 [12.7 (7.3–20.1)]	9 [7.6 (3.5–13.9)]	4 [3.4 (0.9–8.5)]
10–12	93 (25.6)	31 [33.3 (25.8–46.1)]	11 [11.8 (6.1–20.2)]	3 [3.2 (0.6–9.1)]	4 [4.3 (1.2–10.5)]
13–16	152 (41.9)	33 [21.7 (15.4–29.1)]	11 [7.2 (3.7–12.6)]	7 [4.6 (1.9–9.3)]	2 [1.3 (1.9–9.3)]
Total	363 (100)	105 [28.9 (24.3–33.9)]	39 [10.7 (7.8–14.4)]	19 [5.2 (3.2–8.1)]	10 [2.8 (1.3–5.0)]
*Gender*					
Male	159 (43.8)	46 [28.9 (22.0–36.6)]	19 [11.9 (7.4–18.0)]	10 [6.3 (3.1–11.3)]	8 [5.0 (2.2–9.7)]
Female	204 (56.2)	59 [28.9 (22.8–35.7)]	29 [14.2 (9.7–19.8)]	9 [4.4 (2.0–8.2)]	2 [1.0 (0.1–3.5)]
*Study sites*					
Bushulo	232 (63.9)	69 [29.7 (23.9–36.1)]	26 [11.2 (7.5–16.0)]	18 [7.8 (4.7–12.0)]	6 [2.6 (1.0–5.5)]
Finchawa	64 (17.6)	18 [28.1 (17.6–40.8)]	4 [6.3 (1.7–15.2)]	1 [1.6 (0.0–8.4)]	3 [4.7 (1.0–13.1)]
PTC	67 (18.5)	18 (26.9, 16.8–39.1)	9 [13.4 (6.3–24.0)]	0 (0.0)	1 [1.5 (0.0–8.0)]

*CI* Confidence interval, *PTC* St Paul’s Tullo Catholic school

### Helminth detection rates by DM and FECT across ages and their diagnostic agreement

**Fig. 2:**
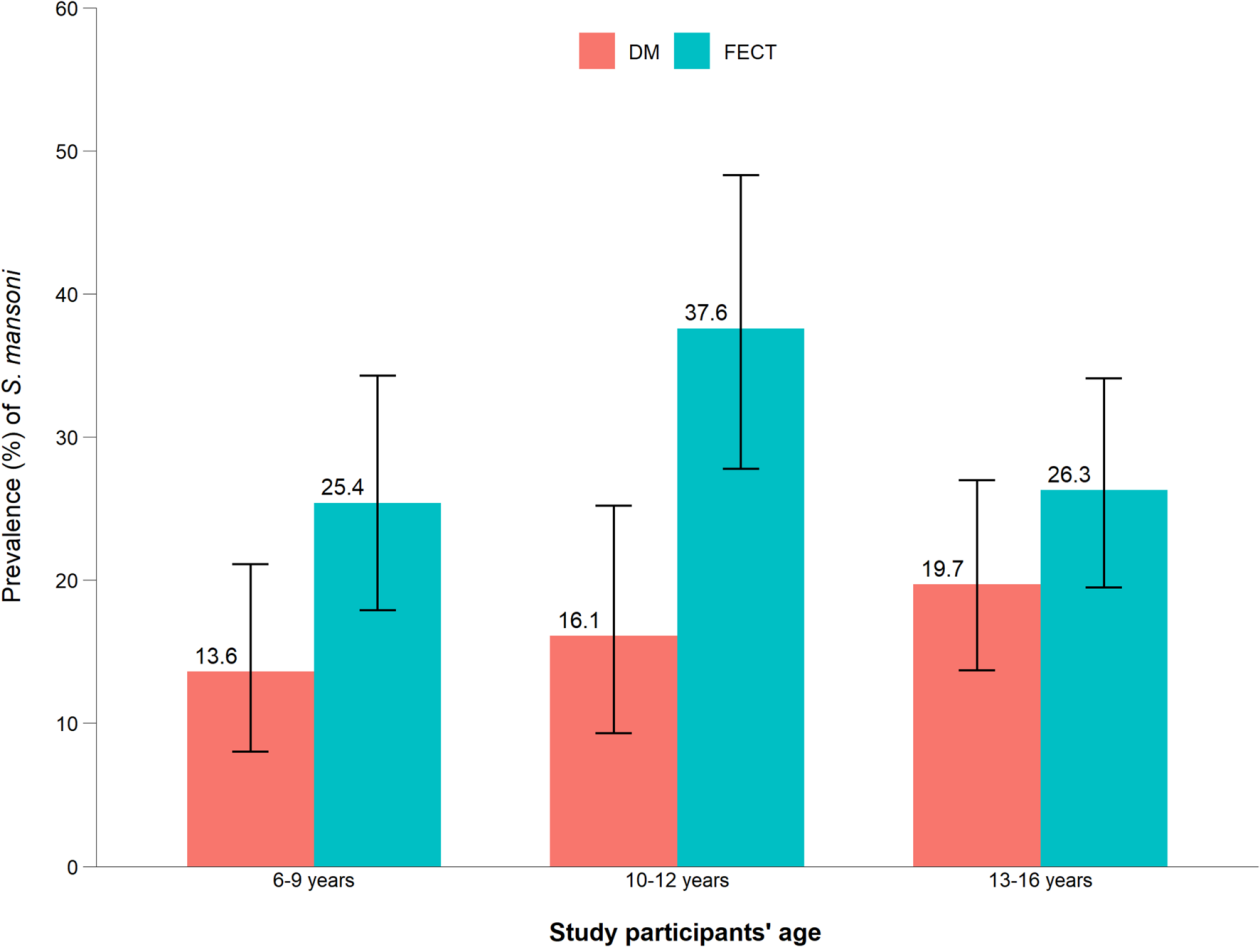
Prevalence of *Schistosoma mansoni* infection, with 95% confidence intervals, across different age groups using DM and FECT in three rural primary schools, Hawassa, southern Ethiopia, 2023. DM, Direct wet mount microscopy; FECT, formol-ether concentration technique

**Fig. 3:**
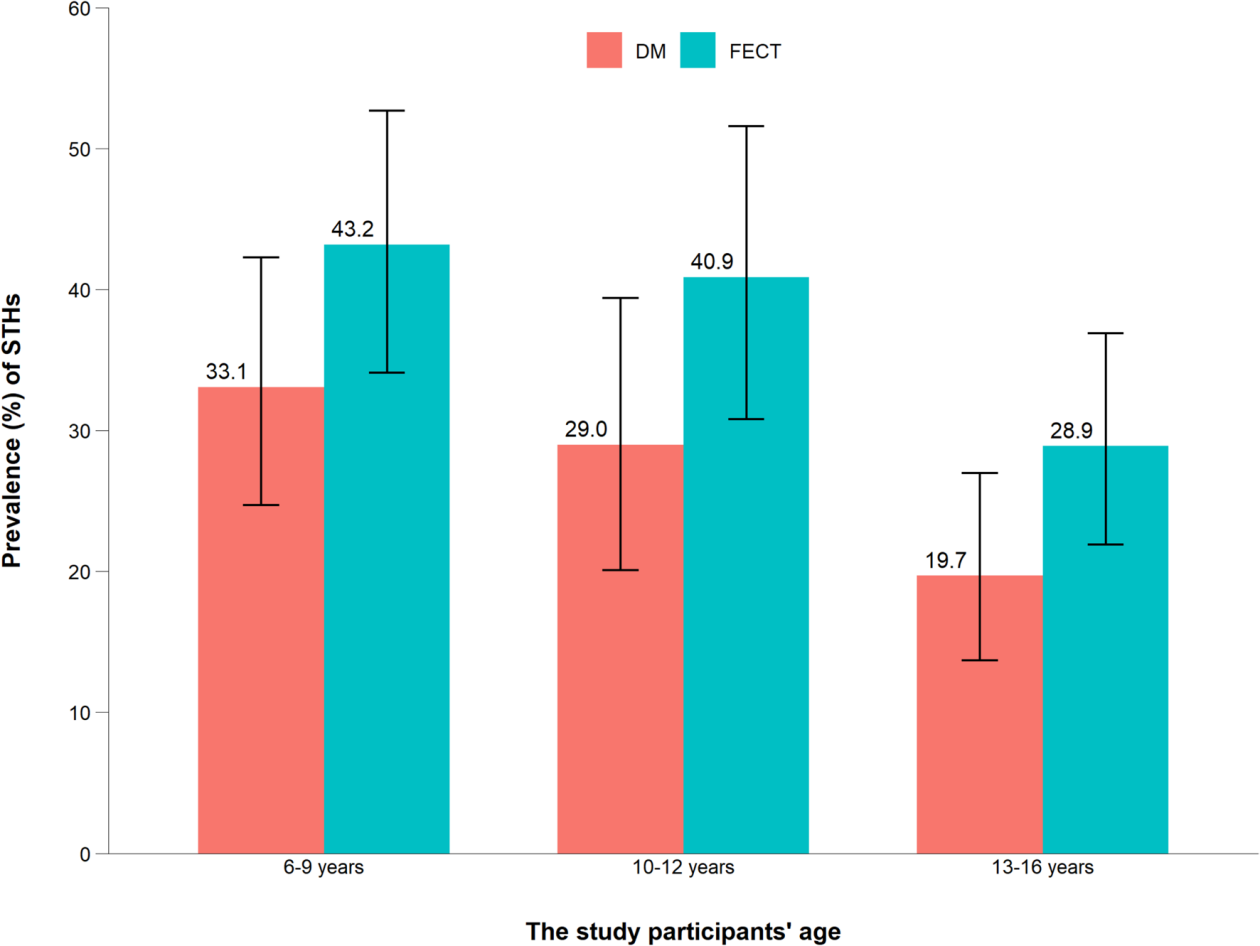
Prevalence of STHs, with 95% confidence intervals, across different age groups using DM microscopy and FECT in three rural primary schools, Hawassa, southern Ethiopia, 2023. DM, Direct wet mount; FECT, formol-ether concentration technique; STHs, soil-transmitted helminths

Figures 2 and 3 show the detection rates of *S. mansoni* and STHs across different age groups using DM microscopy and FECT. The detection rate of *S. mansoni* by FECT was 2.3-fold higher than that by DM microscopy in the age group 10–12 years, 1.9-fold higher in the age group 6–9 years and 1.3-fold higher in the age group 13–16 years. For STHs, the detection rate by FECT was 1.3-, 1.4- and 1.5-fold higher than that by DM microscopy in the age groups 6–9, 10–12 and 13–16 years, respectively.

The diagnostic agreement between DM microscopy and FECT for detecting helminth infections is shown in Table 5. The results for Cohen’s *κ* indicate substantial agreement (*κ* = 0.710) between DM microscopy and FECT for detecting *H. nana* ova and almost perfect agreement (*κ* = 0.827) for detecting *A. lumbricoides* ova. The agreement between the two diagnostic methods was fair, with *κ* values ranging from 0.273 to 0.399, for detecting *T. trichiura*, *Taenia* species, *S. mansoni, E. vermicularis* and hookworm ova.

**Table 5:** Diagnostic agreement between direct wet mount microscopy and the formol-ether concentration technique for the detection of helminth ova among schoolchildren from three rural schools in Hawassa, southern Ethiopia, 2023

Direct wet mount microscopy	Formol-ether concentration technique			
	Positive test result	Negative test result	Type of parasite	*κ* (95% CI)
			Hookworm	
Positive test result	5	3		0.399 (0.15–0.65)
Negative test result	11	344		
			*Ascaris lumbricoides*	
Positive test result	81	3		0.827 (0.76–0.89)
Negative test result	21	258		
			*Trichuris trichiura*	
Positive test result	7	3		0.273 (010–0.44)
Negative test result	29	324		
			*Schistosoma mansoni*	
Positive test result	43	18		0.388 (0.28–0.49)
Negative test result	62	240		
			*Enterobius vermicularis*	
Positive test result	2	4		0.392 (0.01–0.78)
Negative test result	2	355		
			*Hymenolepis nana*	
Positive test result	9	2		0.710 (0.51–0.91)
Negative test result	5	346		
			*Taenia* species	
Positive test result	1	1		0.396 (-0.14–0.94)
Negative test result	2	359		

* CI* Confidence interval, *κ* Cohen’s kappa

## Discussion

We found that the overall prevalence of intestinal helminthic infections among the schoolchildren included in our study was 59.8%, with 33.9% and 38.8% of children infected with *S. mansoni* and STHs, respectively. The STHs identified were *A. lumbricoides* (28.9% infected children), *T. trichiura* (10.7%) and hookworms (5.2%). About 25% of the children had co-infections. STH infections were more common among children who sometimes (vs always) washed their hands before meals and those who had a habit of playing with soil. Additionally, children at Finchawa Primary School had a lower prevalence of *S. mansoni* infection compared to those at PTC.

The observed prevalence of helminth infections (59.8%, 95% CI 54.5–64.9) in the current study was consistent with previous findings (67.9%, 95% CI 62.9–72.6) from the study schools in 2015 [10]. However, in contrast to the previous study’s result (52.7%, 95% CI 47.2–57.6) in the schools [10], a lower prevalence of STHs (38.8%, 95% CI 33.8–44.1) was observed in the present study. As there was no difference in the magnitude of *T. trichiura* (11%, 95% CI 8.0–14.6) and hookworm infections (7.7%, 95% CI 5.3–11.0) [10] between the two studies, the observed decrease in STHs was attributed to a reduction in *A. lumbricoides* prevalence from 44.4% (95% CI 39.3–49.6) in 2015 [10] to 28.9% (95% CI 24.3–33.9) in the current study. Our results were also comparable with recent findings on STHs from Ethiopia: infections with *A. lumbricoides* (27.4%) and hookworms (4.3%) in north-central Ethiopia [19], *T. trichiura* (7.8%) and hookworm infections (5.5%) in northwest Ethiopia [20], and hookworm infection (4.4%) in southern Ethiopia [21]. However, contrasting data were reported from other areas in Ethiopia, with a higher prevalence of hookworms (78.7%) and a lower prevalence of *A. lumbricoides* (2.5%) and *T. trichiura* (0.6%) [3].

The observed decrease in the prevalence of STHs, particularly in *A. lumbricoides* infections, which remained comparable in 2015 and 2007 [10, 11], indicates the significance of control interventions. Given that *A. lumbricoides* significantly contributes to soil pollution and sustains its transmission cycle due to factors such as worm load, fertility and the eggs' resistance to environmental stress [22], intensifying deworming interventions is essential. Enhancing deworming efforts for adults could also help reduce the reinfection of children. Although mebendazole, an antiparasitic medication used by the Ethiopian national program, is effective against *A. lumbricoides*, its much lower cure rates for *T. trichiura* and hookworms need to be addressed for better control of these infections [23]. Our findings underscore the importance of educating and assisting children in improving hand-washing before meals and avoiding playing with soil.

The prevalence of *S. mansoni* infection (33.9%; 95% CI 29.0–39.0) in the current study is consistent with the results from 2015 (31%) [10], although it represents a substantial decline from the prevalence (76.5%) in 2007 [11]. The lower prevalence of *S. mansoni* infection in children at the Finchawa school compared to the PTC school is in contrast with the 2015 findings, which showed the reverse trend. This reversal in trend may be linked to changes in the children’s interactions with the lake. *Schistosoma mansoni* infection remains a public health concern among schoolchildren, underscoring the need to address gaps in intervention implementation, particularly in terms of ensuring the regular provision of preventive chemotherapy. The regional health bureau should prioritize making praziquantel more widely available, especially to high-risk children residing near Hawassa Lake. The prevalence observed in the present study is considerably higher than that reported recently from Guangua District, northwest Ethiopia (12.6%) [24], Guder town, west Ethiopia (3.6%) [25] and Sasiga District, southwest Ethiopia (4.4%) [26], indicating the varying significance of this infection in different localities.

In areas where various helminthic infections are prevalent, co-infections are common. A considerable proportion of the children in the present study (23.1%; 95% CI 18.9–27.8) harbored co-infections, consistent with the values reported in 2015 (25.7%) [10]. The most common co-infection was a dual infection with *A. lumbricoides* and *S. mansoni* (7.4%), likely due to their higher prevalence, which favors their concurrence. Co-infections involving helminths from different transmission pathways may suggest a severe lack of WASH access in the community, which can be difficult to detect at the household level due to variable access [27]. Thus, together with consistent deworming interventions, improving community-wide WASH access is crucial to reducing helminth prevalence.

Regarding clinical or research purposes, it is recommended to examine several stool specimens collected over consecutive days to maximize the sensitivity of parasitological tests and reduce the rate of incorrect diagnosis [28]. However, it may be impractical to perform multiple stool examinations for every individual in clinical settings with high patient loads. The detection rate is lower when based on a single stool examination, and employing a low-sensitive method in clinical settings can further undermine the diagnosis of helminth infections. The use of a method like FECT, which is rapid and can be used over a wide range of concentrations of helminth parasites [28, 29] should be considered in clinical settings. In the present study, the results of DM microscopy were in almost perfect agreement with those of FECT in terms of diagnosing ova of *A. lumbricoides* (*κ* = 0.827), consistent with a previous study [13]. This agreement is likely related to the large number of *A. lumbricoides* eggs excreted in the stool, increasing the chance of detection by DM microscopy, and reflects the significance of employing DM in clinical settings where FECT is inapplicable. For parasites such as *S. mansoni*, *T. trichiura*, *Taenia* species and hookworms, the observed fair degree of agreement (*κ* < 0.40) between the two methods suggests that DM microscopy is less reliable for diagnosing these parasites [13, 22]. Using FECT in clinical settings instead of DM microscopy not only enhances patient management but also strengthens passive surveillance for the early detection of emerging and re-emerging helminth infections in a given geographical area. This approach provides baseline evidence to initiate mapping infection and disease epidemiology. Additionally, leveraging FECT is important in scenarios where the focus is on monitoring infection prevalence rather than morbidity assessment via KK, such as in relatively low transmission areas where the aim is to further reduce infection prevalence [30, 31].

This study has several limitations. First, analyzing only a single stool sample may have influenced the detection rate and underestimated helminth prevalence in the study population. Second, using laboratory techniques like KK could have provided parasite intensity estimates to assess the effectiveness of deworming. Third, comparing results across studies is challenging due to differences in or combined use of parasitological methods. Fourth, the prevalence of some parasites may have been underestimated due to the inability to employ methods such as the Bearman technique for *S. stercoralis* and the Scotch tape method for *E. vermicularis*. Fifth, our assessment of factors related to *S. mansoni* infection did not consider children's engagement with the lake, potentially overlooking important factors. Lastly, since the study was conducted in areas where there is a higher risk for *S. mansoni* infection, the reported prevalence may be higher than it would have been with a random sampling of *kebeles*. Also, the prevalence of STHs may differ in other *kebeles* within the sub-city or other sub-cities, as the study schools did not represent all of the schools in Hawassa.

## Conclusions

This study showed a reduction in STH infections from a high prevalence in 2015 to a moderate prevalence in 2023, primarily due to a decline in ascariasis. However, the moderate prevalence of *S. mansoni* infection observed in 2015 remained unchanged. Therefore, ensuring the implementation of treatment of school-aged children every 2 years for *S. mansoni* and once every year for STHs, as advised by the WHO [32], would help to reduce the burden of infections in the study area. Additionally, improving sanitation and water supply and providing health education would help to sustain the impact of deworming interventions. In clinical settings where DM microscopy is often used, it is imperative to consider employing FECT for helminth diagnosis.

## Supplementary Information

**Supplementary Material 1.**

## Data Availability

Data supporting the main conclusions of the study are provided within the manuscript.

## Abbreviations

AOR: Adjusted odds ratio
CI: Confidence interval
DM: Direct wet mount
FECT: Formol-ether concentration technique
KK: Kato-Katz
PTC: St Paul’s Tullo Catholic (primary school)
STHs: Soil-transmitted helminths
